# Etiologie rare d’un syndrome subocclusif: polype fibrinoïde inflammatoire de l’iléon, à propos d’un cas clinique

**DOI:** 10.11604/pamj.2017.26.146.10775

**Published:** 2017-03-14

**Authors:** Redouane Ahtil, Mustapha bensghir, Mohammed Meziane, Abdelhafid Houba, Abelhamid Jaafari, Salim Jaafar Lalaoui, Charki Haimeur

**Affiliations:** 1 Service d'Anesthésiologie, Hôpital Militaire Med V, Université Souissi Med V, Rabat, Maroc

**Keywords:** Polype fibroïde inflammatoire, iléon, syndrome subocclusif, chirurgie, Inflammatory fibroid polyp, ileum, subocclusive syndrome, surgery

## Abstract

Le polype fibroïde inflammatoire (PFI) est une lésion bénigne rare, qui découle de la sous-muqueuse du tractus gastro-intestinal, apparait généralement comme une lésion bénigne solitaire, localisée rarement au niveau de l'iléon. Son origine est controversée. La présentation clinique varie selon sa localisation, l'invagination et l'obstruction constituent les symptômes révélateurs les plus fréquents quand le polype siège au niveau de l'intestin grêle. Nous rapportons le cas d'un patient âgé de 22 ans, qui présentait une douleur abdominale, des nausées et des vomissements avec des antécédents de constipation intermittente et une perte de poids dans l'année précédente. La radiologie a objectivé une invagination iléo-iléale obstruant complètement la lumière de l'iléon. La résection segmentaire du segment iléal obstrué et l'anastomose termino-terminale ont été effectuées. Le diagnostic final de PFI a été établi par l'examen histologique et immuno-histochimique.

## Abstract (en)

Inflammatory fibroid polyp (IFP) is a rare benign lesion, originating from the submucosa in the gastrointestinal tract. It generally appears as an isolated benign lesion, rarely located at the level of the ileum. Its origin is controversial. Clinical presentation varies depending on its location; invagination and obstruction are the most common indicative symptoms when the polyp is located at the level of the small intestine. We report the case of a 22-year old patient with abdominal pain, nausea and vomiting and a personal history of intermittent constipation and a weight loss during the previous year. Radiological imaging objectified ileo-ileal invagination completely obstructing the ileum light. Segmental resection of the obstructed ileal segment and termino-terminal anastomosis were performed. The final diagnosis of IFP was established using histological examination and immunohistochemical investigation.

## Introduction

Le polype fibroïde inflammatoire ou polype fibro-inflammatoire est une lésion bénigne très rare qui découle de la sous-muqueuse du tractus gastro-intestinal, le plus souvent dans l'antre gastrique (70%) et l'iléon (20%) et occasionnellement, dans le duodénum et le jéjunum [1, 2]. Il s'observe essentiellement chez l'adulte et très occasionnellement chez l'enfant [1]. La symptomatologie clinique et l'imagerie diffèrent d'une localisation à l'autre, mais au niveau intestinal, l'invagination comme mode de révélation souvent rapportée chez l'adulte [3]. Nous rapportons l'observation du polype fibroïde inflammatoire de l'iléon révélé par une invagination iléo-iléale.

## Patient et observation

**Figure 1:**
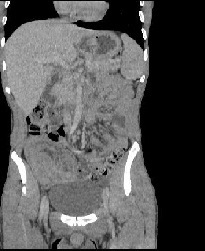
TDM montrant une masse de la fosse iliaque droite avec un aspect en pseudo-rein

**Figure 2:**
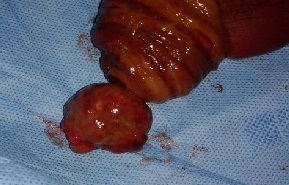
Polype pédiculé de 3x3x3 cm

**Figure 3:**
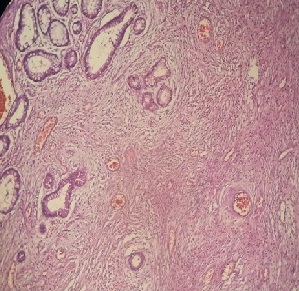
Prolifération muqueuse et sous muqueuse de cellules fusiformes disposées en faisceaux lâche

Il s'agit d'un jeune patient de 22 ans présentait des douleurs abdominales, des vomissements et des nausées. Il avait des antécédents de constipation intermittente associée à une perte de poids dans l'année précédente. Le cliché radiologique d'abdomen sans préparation a montré des segments dilatés de l'intestin grêle avec des niveaux hydro-aériques marqués. La tomodensitométrie abdomino-pelvienne a objectivé une masse dans la fosse iliaque droite avec un aspect en pseudo-rein en faveur d'une invagination intestinale (Figure 1). La laparotomie a révélé une invagination iléon-iléale obstruant complètement la lumière de l'iléon. La résection segmentaire du segment iléal obstrué et l'anastomose termino-terminale ont été effectuées. L'examen macroscopique de la pièce a montré un polype mesurant 3x3x3 cm se projetant dans la lumière de l'iléon (Figure 2). L´examen microscopique : prolifération au niveau de la muqueuse et sous-muqueuse de cellules fusiformes disposées en faisceaux lâches ou des structures courtes verticillées, généralement disposées en « pelure d'oignon » autour des capillaires abondants avec un infiltrat inflammatoire abondant associé dominé par les éosinophiles (Figure 3).

## Discussion

Le polype fibroïde inflammatoire (PFI) est une lésion rare et bénigne du tube digestif [1]. Cette tumeur est découverte à tout âge, mais est plus souvent chez l´adulte surtout entre 60 et 70 ans [1]. La localisation gastrique est de loin la plus fréquente. Elle constitue 70% des cas et intéresse le plus souvent l'antre. La localisation grêlique est plus rare. Elle représente 18 % des cas et prédomine au niveau du jéjunum. Les localisations iléale et duodénale sont plus rares [4]. La particularité de cette lésion, lorsqu'elle est localisée au niveau du grêle, comme le cas de notre patient, est d'entraîner une invagination intestinale aigüe [5]. La symptomatologie clinique est polymorphe et le plus souvent trompeuse: tableau occlusif aigu, tableau subocclusif de survenue progressive s'étendant de quelques jours à quelques semaines, syndromes abdominaux non spécifiques (modification du transit, douleurs abdominales diffuses, saignements digestifs?), évoluant parfois pendant plusieurs mois, avec ou sans altération de l'état général. Selon les auteurs l'invagination intestinale chez l'adulte est rarement une cause d'occlusion vraie mais plutôt de subocclusion douloureuse résolutive [3]. Le diagnostic para-clinique est surtout radiologique, la radiographie de l'abdomen sans préparation peut aider dans le diagnostic de l'invagination intestinale. Si l'invagination intestinale se présente avec des signes d'occlusion aigue, les clichés vont mettre en évidence des images hydro-aériques souvent localisées. Ces images sont de forme et de topographie variables suivant le siège de l'invagination. La radiographie en double contraste a fait la preuve de sa supériorité pour le diagnostic de petites lésions digestives. Le lavement baryté standard a une efficacité diagnostique variable selon la qualité de la préparation et de l'examen, il ne détecte que les lésions de 1 cm et plus. Le transit du grêle longtemps resté l'examen de référence pour l'exploration des zones de l'intestin qui étaient jusqu'à lors inaccessibles à l'endoscopie (entre la première anse jéjunale et l'iléon terminal). Le transit du grêle est d'interprétation bien délicate, même lorsque les conditions d'une bonne exploration radiologique sont réunies ; il existe de nombreux faux positifs : artéfacts liés à la présence d'un contenu endoluminal solide ou gazeux, spasmes, compressions extrinsèques par les organes de voisinage. Aussi, la sensibilité globale de cet examen pour la détection des polypes non occlusifs ou non ulcérés est-elle très insuffisante. Le PFI du grêle apparaît sous forme d'une image de soustraction plus ou moins pédiculée. La tomodensitométrie largement indiquée chez l'adulte, Il permet de faire le diagnostic positif de l'invagination et de décrire, à la limite du possible, la lésion causale (siège, densité spontanée et après injection de produit de contraste, rapports aux structures avoisinantes). Dans notre cas, la tomodensitométrie a montré une masse dans le quadrant inférieur droit avec un aspect en pseudo-rein et a suggéré une invagination intestinale, sans pouvoir en préciser l'étiologie. La recherche de signes de gravité est essentielle pour la prise en charge et le pronostic. Le scanner est le meilleur examen pour prédire de la souffrance intestinale. L'invagination intestinale devient un diagnostic urgent lorsqu'il existe une strangulation avec souffrance ischémique de l'anse concernée [6]. L'entéroscanner, par son excellente résolution spatiale, sa reproductibilité, au prix d'une irradiation souvent non négligeable, est un examen de choix pour l'étude des lésions transmurales ou extramurales, tumorales et inflammatoires [7]. L'entéro-IRM a l'avantage d'être non irradiante mais ses performances sont moins bonnes que l'entéroscanner pour la détection des polypes du grêle. L'endoscopie est le seul examen qui permet de visualiser théoriquement la totalité du tube digestif, et de réaliser une biopsie ou l'exérèse des lésions observées. L'endoscopie représente l'examen de référence pour la détection, la classification et la surveillance des polypes. L'entéroscopie poussée est surtout utilisée par voie haute, car l'exploration par voie basse ne semble pas plus efficace qu'une iléo-coloscopie standard. Elle peut également être utile pour faire des biopsies des lésions repérées au préalable par la vidéo-capsule. Du point de vue anatomie pathologique Les polypes fibroides sont des lésions qui naissent de la sous muqueuse. Son expansion est intra-luminale de type sessile surtout dans l´estomac ou pédiculé dans l'iléon. Le diamètre varie de quelques millimètres à plusieurs centimètres, les lésions gastriques étant généralement inférieures au centimètre et les lésions iléales dépassant 2 cm [8]. Elle atteint rarement plus de 6 cm [8], mais il y a un cas de PFI rapporté avec 12,5 cm de diamètre [8]. La tumeur est unique quoique quatre cas de lésions multiples du tractus digestif aient été décrits [8]. A la section, elle apparait ferme, homogène, pâle (blanc grisâtre ou blanc jaunâtre), brillante, parfois myxoïde. La section confirme l´origine sous- muqueuse. Microscopiquent le polype est constitué par un tissu fibro-inflammatoire richement vascularisé où s´associent, dans des proportions variables, trois contingents: fibroblastique, vasculaire et inflammatoire. L´infiltrat inflammatoire est toujours présent. Les polynucléaires neutrophiles sont rares. Les éosinophiles (en quantité variable) prédominent habituellement mais on rencontre aussi un contingent de lymphocytes, plasmocytes et mastocytes dans tous les cas. Les lymphocytes peuvent former des amas en l´absence de centres germinatifs. Ces tumeurs proches des pseudotumeurs inflammatoires s'en différencient par une taille plus petite, une actine peu souvent positive et un CD 34 + (82%) [111] et ALK1 [9]. L'entité histologique actuellement appelée polype fibro-inflammatoire (PFI) est non seulement caractérisée par des mutations génétiques, observées dans un grand pourcentage de cas, mais quelques cas peuvent être également associés à des infections ou un processus immunologique, ce qui suggère différentes approches thérapeutiques [10]. La plupart des polypes fibroides peuvent être traités par voie endoscopique. La chirurgie est rarement nécessaire. Le patient peut, cependant, souffrir de graves perturbations dues aux saignements des muqueuses, l´obstruction locale ou l'invagination intestinale. Le traitement est certainement chirurgical dans les formes intestinales, surtout celles compliquées d´invagination. Dans notre cas, l'invagination a été réduite spontanément avant l'intervention et une résection segmentaire de l'intestin a pu être réalisée.

## Conclusion

Le polype fibroïde inflammatoire est une lésion rare et bénigne du tube digestif. Il s'observe essentiellement chez l'adulte et très occasionnellement chez l'enfant. Sa pathogénie est inconnue. Il est habituellement découvert fortuitement, son exérèse endoscopique et l'étude anatomo-pathologique permettent de poser le diagnostic de certitude. Bien que les polypes fibroïdes inflammatoires sont très rares, ils sont parmi les diagnostics probables qui devraient être considérés dans les tumeurs obstructives de l´intestin grêle chez l'adulte.
